# Self-delivery photothermal-boosted-nanobike multi-overcoming immune escape by photothermal/chemical/immune synergistic therapy against HCC

**DOI:** 10.1186/s12951-024-02399-3

**Published:** 2024-03-29

**Authors:** Huizhen Yang, Weiwei Mu, Shijun Yuan, Han Yang, Lili Chang, Xiao Sang, Tong Gao, Shuang Liang, Xiaoqing Liu, Shunli Fu, Zipeng Zhang, Yongjun Liu, Na Zhang

**Affiliations:** NMPA Key Laboratory for Technology Research and Evaluation of Drug Products, Department of Pharmaceutics, Key Laboratory of Chemical Biology (Ministry of Education), School of Pharmaceutical Sciences, Cheeloo College of Medicine, Shandong University, 44 Wenhua Xi Road, Jinan, 250012 Shandong China

**Keywords:** Immune checkpoint inhibitors, Black phosphorus, Tumor immunosuppressive, Immune escape, Anti-vascular therapy, Hepatocellular carcinoma

## Abstract

Immune checkpoint inhibitors (ICIs) combined with antiangiogenic therapy have shown encouraging clinical benefits for the treatment of unresectable or metastatic hepatocellular carcinoma (HCC). Nevertheless, therapeutic efficacy and wide clinical applicability remain a challenge due to “cold” tumors’ immunological characteristics. Tumor immunosuppressive microenvironment (TIME) continuously natural force for immune escape by extracellular matrix (ECM) infiltration, tumor angiogenesis, and tumor cell proliferation. Herein, we proposed a novel concept by multi-overcoming immune escape to maximize the ICIs combined with antiangiogenic therapy efficacy against HCC. A self-delivery photothermal-boosted-NanoBike (BPSP) composed of black phosphorus (BP) tandem-augmented anti-PD-L1 mAb plus sorafenib (SF) is meticulously constructed as a triple combination therapy strategy. The simplicity of BPSP's composition, with no additional ingredients added, makes it easy to prepare and presents promising marketing opportunities. (1) NIR-II-activated BPSP performs photothermal therapy (PTT) and remodels ECM by depleting collagen I, promoting deep penetration of therapeutics and immune cells. (2) PTT promotes SF release and SF exerts anti-vascular effects and down-regulates PD-L1 via RAS/RAF/ERK pathway inhibition, enhancing the efficacy of anti-PD-L1 mAb in overcoming immune evasion. (3) Anti-PD-L1 mAb block PD1/PD-L1 recognition and PTT-induced ICD initiates effector T cells and increases response rates of PD-L1 mAb. Highly-encapsulated BPSP converted 'cold' tumors into 'hot' ones, improved CTL/Treg ratio, and cured orthotopic HCC tumors in mice. Thus, multi-overcoming immune escape offers new possibilities for advancing immunotherapies, and photothermal/chemical/immune synergistic therapy shows promise in the clinical development of HCC.

**Graphical Abstract:**

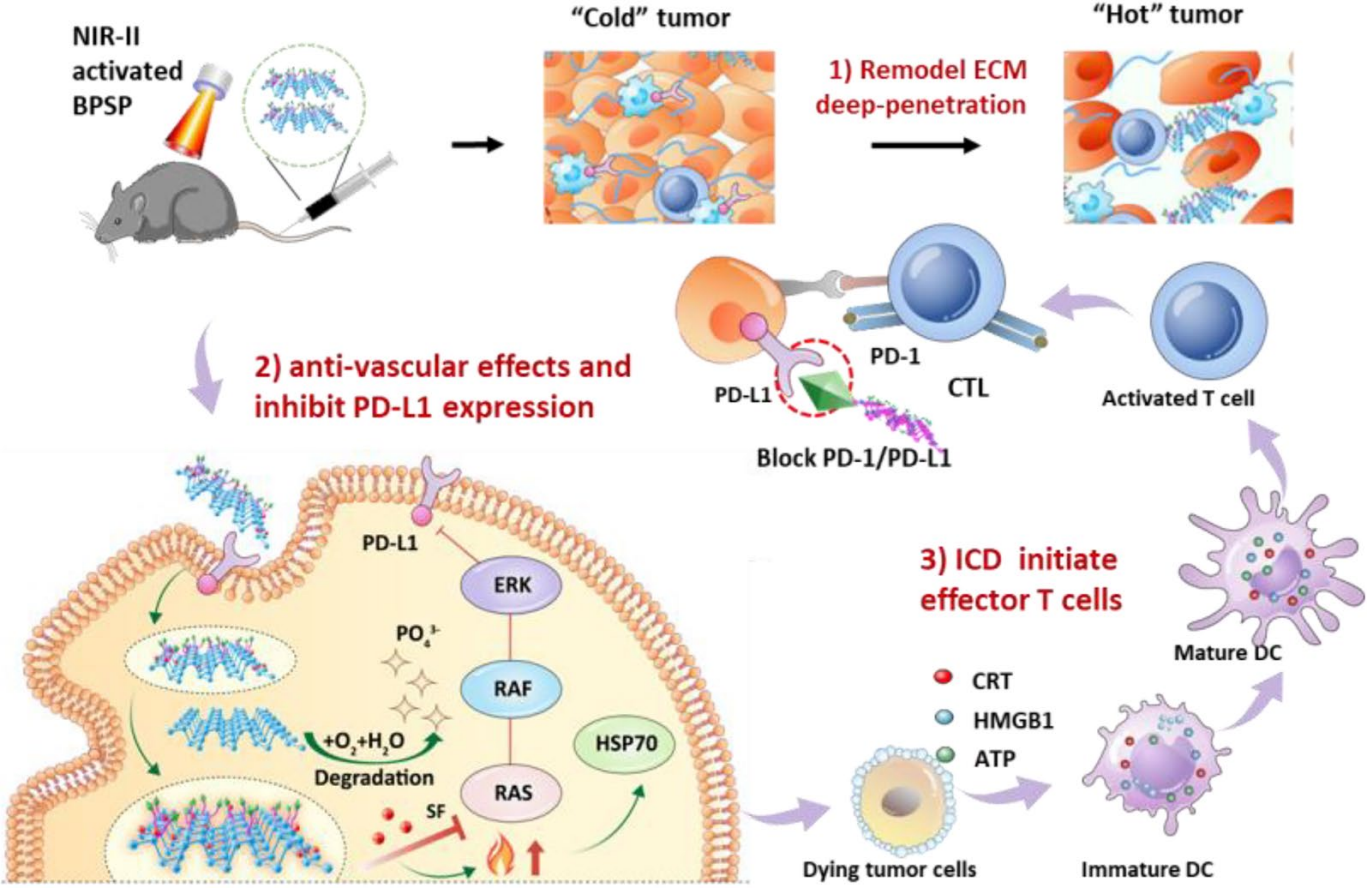

**Supplementary Information:**

The online version contains supplementary material available at 10.1186/s12951-024-02399-3.

## Introduction

Hepatocellular carcinoma (HCC) seriously threatens human health worldwide as one of the most common malignant tumors [1]. Due to the high immunotolerance of the liver, HCC typically exhibits a ‘cold’ tumor phenotype with a complex tumor immunosuppressive microenvironment (TIME) [2]. Accordingly, the conversion of HCC from ‘cold’ immunologically inactive tumors to ‘hot’ ones would be a promising strategy for tumor immunotherapy. Among them, immune checkpoint inhibitors (ICIs) are the most prominent immunotherapeutic, which has propelled the HCC treatment into a new era [3,4]. For instance, the PD-1/PD-L1 signaling pathway is the most widely studied and a series of products have been approved for the market [5,6]. However, the major drawback of ICIs lies in their low responsiveness, with a large proportion of patients unable to successfully activate their intrinsic immune system, resulting in treatment failure [7,8]. This is actually owing to that complex signal crosstalk exists between the components in TIME [9], a continuously natural driving force for immune escape by extracellular matrix (ECM) infiltration, tumor angiogenesis, and tumor cell proliferation [10,11]. Consequently, targeting ICIs alone is far from sufficient for tumor immunotherapy, and only if comprehensive effects of TIME on immunosuppression are effectively blocked can the HCC treatment be successful.

The main reason of TIME on immune escape could be summarized into three aspects: Firstly, the presence of abundant ECM in HCC plays a crucial role in impeding the anti-tumor immune response. The dense barriers of ECM not only prevent the infiltration of immune cells into the tumor but also induce the polarization of TAMs towards the immunosuppressive M2 phenotype, which promotes the up-regulation of PD-L1 levels [12–14]. Secondly, tumor vascular endothelial cells up-regulate the expression of immune checkpoint by secreting VEGF, which causes the invalidation of T cells [15]. Herein, the use of angiogenesis inhibitors is considered to be an effective approach to improve the efficacy of ICIs. The combination treatment of atezolizumab plus bevacizumab (anti-PD-L1 mAb plus anti-vascular drug) was shown to provide superior outcomes and has become the new standard first-line treatment for unresectable or metastatic HCC [16]. Thirdly, immune checkpoint protein is highly expressed on tumor cells, which avoids the recognition by immune cells through continuous variation, expression of antigen-degrading enzymes and release of immunosuppressive factors [17]. In particular, high expression of PD-L1 on tumor-associated macrophages (TAMs) inhibits T cell activity [18,19]. Aberrant accumulation of PD-L1 highly expressed TAMs in liver tissues further facilitates immune escape. Therefore, simultaneously eliminating the immune escape generated by ECM, tumor angiogenesis, and tumor cells, would be an important prerequisite for the success of HCC immunotherapy.

To achieve this goal, multi-overcoming immune escape by photothermal/chemical/immune combination regimen was proposed. Photothermal treatment (PTT) is a physical and non-invasive tumor treatment pattern with widespread application in clinics. PTT not only kills the tumor cells but also remodels ECM by reducing collagen-1 expression, thus facilitating the deep penetration of immune cells. Furthermore, PTT induces immunogenic cell death (ICD), which robustly triggers the antitumor immune responses required for rebalancing the TIME [20,21]. Black phosphorus (BPs) is the most prospective two-dimensional (2D) layered inorganic material with excellent NIR-II photothermal conversion efficiency, high drug loading capacity, and good biocompatibility [22–26], it could be utilized as the PTT photosensitizer and self-delivery drug nanocarriers. Chemotherapy is an indispensable clinical treatment means, and choosing the appropriate drug, which could specifically target the high-expressed receptors on both tumor cells and tumor vascular endothelial cells, would be a feasible approach to relief the immunosuppression. Sorafenib (SF) is the first-line multikinase inhibitor approved by the FDA for HCC treatment, which could suppress tumor cell proliferation by inhibiting Ras/Raf/MEK/ERK signaling pathways, and exert an antiangiogenic effect by targeting VEGFR [27]. Additionally, SF could decrease PD-L1 levels on tumor cells and TAMs by inhibiting the activity of Ras/Raf-MAPK and PI3K-AKT signaling [28], which would rebalance TIME and enhance the therapeutic effect of ICIs. Therefore, the combination of BP and SF was selected in this study to simultaneously kill tumor cells, inhibit tumor angiogenesis, and reduce ECM, which would effectively eliminate immune escape and thus provide a favorable immunotherapeutic condition. On this basis, the biological anti-PD-L1 mAb were further selected to maximize the immunotherapeutic effect of ICIs, thereby activating the immune response. Briefly, the combination of BP/SF/anti-PD-L1 mAb could realize the photothermal/chemical/immune synergistic effects for HCC treatment.

**Scheme 1.**
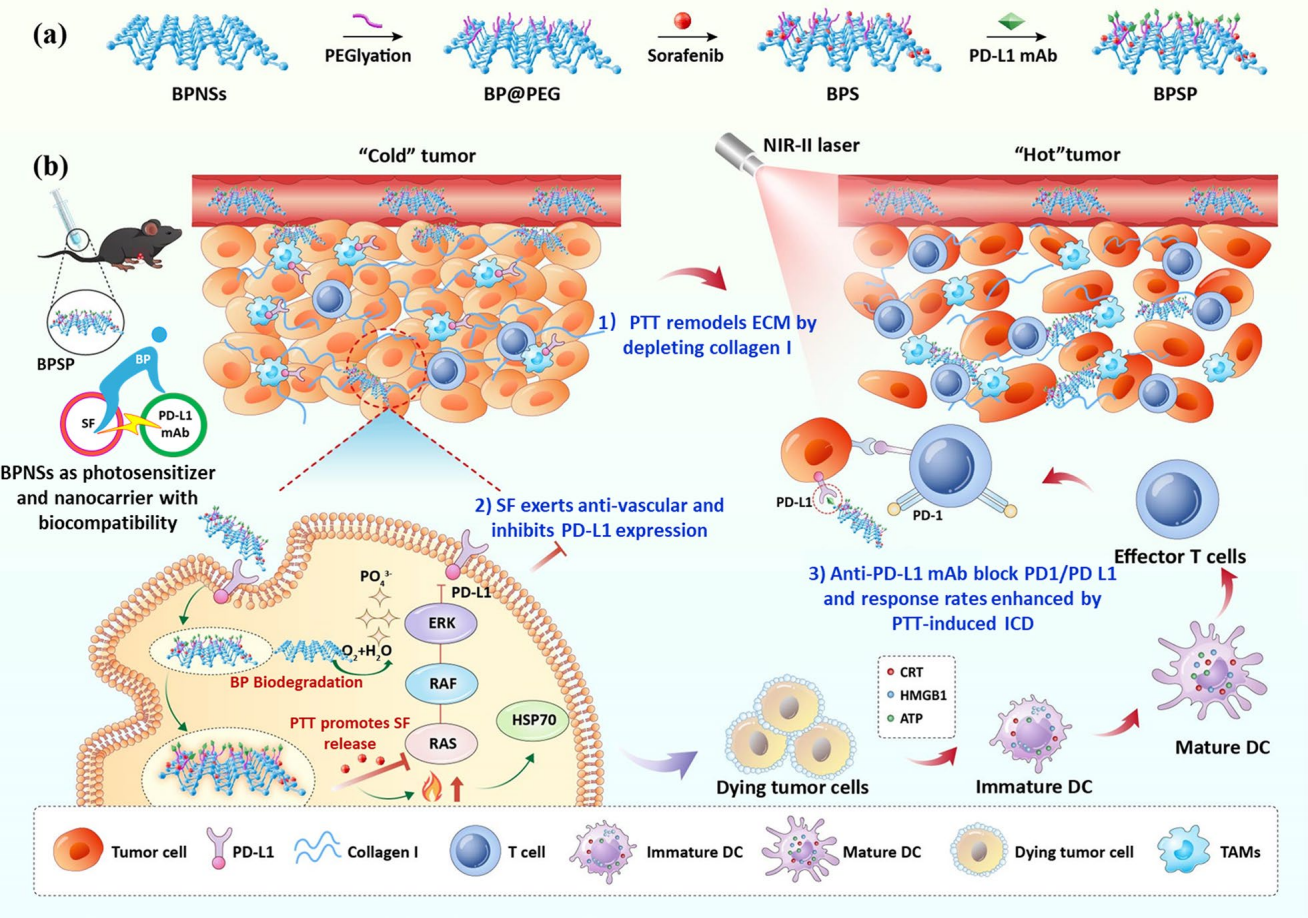
Schematic illustration of triplet combination self-delivery photothermal-boosted-NanoBike (BPSP) tandem-augmented anti-PD-L1 mAb plus SF against HCC. **a** Preparation of BPSP. **b** BPSP multi-overcoming immune escape by simultaneously reducing ECM inhibiting tumor angiogenesis, and inducing ICD, finally photothermal/chemical/immune killing tumor cells. (1) NIR-II-activated BPSP performs PTT and remodels ECM by depleting collagen I, promoting deep penetration of therapeutics and immune cells. (2) SF released from BPSP by PTT promotion, which exerts anti-vascular effects and down-regulates PD-L1 via RAS/RAF/ERK pathway inhibition, enhancing the efficacy of anti-PD-L1 mAb in overcoming immune escape. (3) The anti-PD-L1 mAb block PD-1/PD L1 recognition and the response rates of anti-PD-L1 mAb was further increased by PTT-induced ICD

Due to the huge differences in physical and chemical properties of the three agents, it’s hard to exert the desired therapeutic effect by the free cocktail. In consideration of this, a triplet combination photothermal-boosted-NanoBike (BPSP) was constructed to maximize the efficacy, in which 2D layered BP was utilized as the self-delivery nanocarrier for embedding both SF and anti-PD-L1 mAb (Scheme 1a). BP functions akin to a bicycle bearing in tandem, boosting the efficacy of anti-PD-L1 mAb and synergistically driving their connection through photothermal effects, thereby enhancing the therapeutic potential of the combination. Meanwhile, BP holds promise in the clinic as degradable PTT agents that are easily converted into human health like phosphate and phosphonate [29,30]. BPNSs are used as a carrier and degradable PTT agents, containing SF and PD-L1 mAb, without additional excipients. In addition, it has a convenient preparation process and has the potential for wide application in biomedicine [31,32]. The BPSP multi-overcoming immune escape by simultaneously reducing ECM, inhibiting tumor angiogenesis and black PD-1/PD-L1 recognition, and finally converting 'cold' tumors into 'hot' ones and photothermal/chemical/immune killing tumor cells (Scheme 1b). (1) NIR-II-activated BPSP performs photothermal therapy (PTT) and remodels ECM by depleting collagen I, promoting deep penetration of therapeutics and immune cells. (2) SF released from BPSP by PTT promotion, which exerts anti-vascular effects and down-regulates PD-L1 via RAS/RAF/ERK pathway inhibition, enhancing the efficacy of anti-PD-L1 mAb in overcoming immune escape. (3) The anti-PD-L1 mAb block PD-1/PD L1 recognition and the response rates of anti-PD-L1 mAb was further increased by PTT-induced ICD. These are cleverly combined to achieve combined photothermal/chemical/immune treatment of HCC. In conclusion, the photothermal-boosted-NanoBike plays a photothermal/chemical/immune synergistic therapeutic effect by multi-overcoming immune escape, which holds a promising strategy for HCC treatment.

## Materials and methods

### Materials

Sorafenib was bought from Shanghai Biochempartner Co., Ltd. (Shanghai, RPC). Anti-mouse PD-L1 antibody (Mw = 150kD) was bought from BioLegend (San Diego, USA). Black phosphorus (BP) and Mal-PEG-NH_2_ (Mw = 2kD) were purchased from Beijing hwrk Chemical Co., Ltd. *N*-methyl-2-pyrrolidone (NMP) and Coumarin-6 was obtained from Aladdin Bio-Chem Technology Co. Ltd (Shanghai, China). Mouse HMGB1 ELISA Kit was provided by Solarbio Science & Technology Co., Ltd. (Beijing, China). Western blot antibodies were all provided by Abcam Trading Co., Ltd. (Shanghai, China). ICD-marked antibodies were all provided by Biosynthesis Biotechnology Co., Ltd. (Beijing, China). Cytokine ELISA kits were provided by MultiSciences Biotech Co., Ltd. (Hangzhou, China). All chemicals used are analytical grade.

### Cell lines and animals

Hepa1-6 cells were obtained from the Institute of Immunopharmaceutical Science of Shandong University. Hepa1-6-Luc cells were bought from Hunan Fenghui Biotechnology Co., Ltd. Female C57BL/6 and male C57BL/6 mice (18-20 g/mouse), six-eight weeks, were bought from SPF Biotechnology Co., Ltd. in Beijing.

### Synthesis of BPSP

*Synthesis of BPNSs*: BPNSs were prepared as previously reported [33]. BPNSs were synthesized by dispersing 20 μg BP powder in 50 mL saturated sodium hydroxide solution of NMP, followed by sonication in the ice bath for 6 h. The resulting suspension was centrifuged at 4000 rpm for 20 min, and the supernatant was collected after removing residual unexfoliated particles. Before use, the BPNSs were rotated to remove NMP. *Synthesis of BP@PEG*: PEG-modified BPNSs were prepared by mixing Mal-PEG-NH_2_ with BPNSs at a mass ratio of 4:1 and stirring for 4 h. *Preparation of BP@PEG/SF (BPS)*: 4 mL of 2 mg/mL SF solution was added to 2 mL of 500 µg/mL BP@PEG NSs solution in methanol. Stir the mixture continuously for 4 h, and SF concentration was measured by HPLC. *Preparation of BP@PEG-Anti-PD-L1 mAb (BPP), BP@PEG/SF-Anti-PD-L1 mAb(BPSP)*: The PD-L1 monoclonal antibody (mAb) was thiolated using Traut's reagent in PBS containing 4 mM EDTA and under N_2_ atmosphere for 1 h. The resulting thiolated anti-PD-L1 mAb was purified using a Zeba desalt spin column. BPS or BP@PEG were dispersed in PBS and reacted with thiolated anti-PD-L1 mAb via Michael reaction under an N_2_ environment overnight. Centrifugation was used to remove anti-PD-L1 mAb without modification. Anti-PD-L1 mAb concentration was assessed in the initial solution and supernatant using an enhanced BCA kit (Beyotime Biotechnology, China).

### Characterizations of BPSP

TEM was used to observe the morphology of BPNS and BPSP, while AFM was used to prepare silicon-based samples by the spin-coating method. Measure the particle sizes, PDI, and zeta potentials of BPNSs, BP@PEG, BPS, and BPSP by Zetasizer. The element composition of BPNSs and BPSP was measured using a small-angle X-ray scattering system, while Raman scattering was performed on a Raman micro-spectrometer, and UV/Vis/NIR absorption spectra were recorded at different concentrations. The optical path length absorbance (A/L) was measured by UV at 1064 nm.

### Drug loading and in vitro drug release

BP@PEG (2 mg/mL) was loaded with SF at varying concentrations (2.0–32.0 mg/mL) using a similar procedure to determine drug loading capacity. HPLC was used to measure SF loading (DL%), while BCA assay was employed to quantify PD-L1 mAb.

$${\text{DL}}\% = {\text{W}}_{{\text{loaded drug}}} /\left( {{\text{W}}_{{\text{loaded drug}}} + {\text{W}}_{{{\text{carrier}}}} } \right) \times {1}00\%$$

W-loaded _drug_ and W _carrier_ represented the drug loaded into BPP and BP@PEG systems.

### In vitro drug release

The releasing curve of SF from BPS, BPSP, and BPSP + L groups was investigated using the dialysis bag method. Each dialysis bag contained 1 mL of sample and was placed in a tube with 10 mL release medium (pH 7.4, 1% Tween-80) at 37 ℃ and 100 rpm. The BPSP + L group was irradiated with a 1064 nm laser (0.8 W/cm^2^) for 5 min before the experiment. The released SF was quantified by HPLC.

### In vitro cellular uptake

Hepa1-6 cells (2 × 10^5^ cells/well) were cultured in 12-well plates and treated with medium containing free coumarin 6 (C6), BP@PEG/C6 (BPC), and PD-L1 mAb-BP@PEG/C6 (BPCP) (C6 concentration is 200 ng/mL) for various durations (1, 2, 4 h). The different groups were examined by IFM at specified time points and subsequently analyzed by FCM. The same method was used to compare cellular uptake of BPC and C6-loaded liposomes (Lipo@C6) in Hepa1-6 cells (2 h).

### In vivo imaging and biodistribution

Female C57BL/6 mice bearing Hepa1-6 tumors were used to investigate in vivo imaging and biodistribution through NIRF imaging. IR780 replaced sorafenib to form BP@PEG/IR780 (BPI) and BP@PEG/IR780-PD-L1 (BPIP): 2 mL of 5 mg/mL IR780 solution was added to 2 mL of 500 µg/mL BP@PEG NSs solution in methanol. Stir the mixture continuously for 4 h, and IR780 concentration was measured by UV. BPI or BP@PEG were dispersed in PBS and reacted with thiolated anti-PD-L1 mAb via Michael reaction under an N_2_ environment overnight. Centrifugation was used to remove Anti-PD-L1 mAb without modification. When the tumor grew to approximately 200–500 mm^3^, mice were injected intravenously with 0.1 mL of free IR780, BPI, or BPIP (IR780 concentration of 30 μg/mL). Real-time IVIS spectroscopy was performed at predetermined time points, and the tumor and organs were isolated and analyzed 24 h after administration. A living Image was used to process the results.

### In vitro and in vivo photothermal performance

To determine the in vitro concentration of BPNSs, they were dispersed in 1 mL PBS at concentrations ranging from 20 to 250 μg/mL and irradiated with a NIR-II laser (0.8 W/cm^2^, 5 min). The temperature was monitored with a digital thermometer, and photos were taken with an infrared imager at predetermined time points. Additionally, the photothermal stability of BPNSs, BP@PEG, BPS, and BPSP was evaluated by monitoring dispersion temperatures using the NIR-II laser for 5 min (laser on) and then allowing the temperature to cool to room temperature (laser off) in periodic cycles repeated four times. The temperature of aqueous solutions of the different NSs (BPNSs, BP@PEG, BPS, BPSP) at 0.1 mg/mL was monitored using a digital thermometer at predetermined time points, with water as the control group.

### In vitro cytotoxicity

The toxicity of BPSP was evaluated using MTT assay on Hepa1-6 cells. Cells were seeded at 5 × 10^3^ cells/well in 96-well plates and incubated with varying concentrations of SF (0.1–40 μg/mL) for 48 h. PTT was performed by irradiating cells with NIR-II laser (0.8 W/cm^2^, 3 min), followed by the addition of 20 µL MTT reagent and further incubation for 4 h. Cell viability was determined using a microplate reader (Biotek, USA).

### Evaluation of PD-L1 expression on Hepa1-6 cells and TAMs

FCM was used to assess PD-L1 expression in Hepa1-6 and RAW264.7 cells. After seeding, the cells were treated with PBS, Free SF, BPS, or BPSP solutions for 12 h before collection. The cells were then incubated with PD-L1 and Alexa Fluor 488 antibodies and labeled cells were measured using flow cytometry. PD-L1 expression in Hepa1-6 tumors was examined in vivo after treatment with these solutions, and the tumors were harvested and analyzed after 21 days of treatment. Western blot analysis was used to assess RAS and ERK protein levels in different groups according to the manufacturer's instructions.

### In vivo immunization study

Following in vivo xenograft tumor and orthotopic HCC models, single-cell suspensions were obtained from the tumor or lymph node using copper mesh filtration. Immune cells were obtained through centrifugation, labeled with antibodies, and analyzed via flow cytometry. T, CTL, DC, Treg, and TAM cells were detected, and cytokine levels were measured using an ELISA kit. DCs were analyzed using CD11c-PE, CD80-FITC, and CD86-PerCP antibody staining, while T cells were analyzed using CD3-APC, CD4-FITC, and CD8-PE antibody staining. CTLs were analyzed using anti-CD8-PE and anti-CD3-APC, anti-IFN-γ-AF488 staining, Tregs were analyzed using anti-CD4-PE, anti-CD25-AF488, and anti-Foxp3-AF647 staining, and TAMs were analyzed using PerCP/Cy5.5 anti-F4/80, anti-CD86-AF488, and anti-CD206-APC staining. The serum levels of IL-6, IL-10, IL-12, TGF-β, TNF-α, and IFN-γ in each group were measured using an ELISA kit (Dakewe, Shenzhen, China).

### Biodegradability of NIR-II activated BPSP on collagen I

To investigate the feasibility of NIR-II activated BPSP depleted collagen I in vivo, the collagen I from Hepa1-6 tumors was evaluated after intravenous injection of NS, BPS, and BPSP. Twelve hours after injection, the BPSP + L and BPS + L groups were irradiated for 3 min, and the depletion capability of NIR-II laser irradiation for collagen level by frozen sections was observed.

### Tumor-penetrating ability in vitro and in vivo

A suspension of methylcellulose (0.24%) with 2 × 10^4^ cells/mL (20 µL) was placed on a cell culture dish cover and inverted on a dish containing 5 mL PBS for 5 to 6 days at 37 ℃ and 5% CO_2_. Tumorspheres were collected by pipette and transferred to a 12-well plate with fresh medium containing C6 labeled nanoparticles (C6 = 100 ng/mL). After NIR-II laser (1064 nm) irradiation at 0.8 W/cm^2^ for 3 min, particles were incubated in a cell incubator for 4 h, and then imaged by laser confocal microscopy. The ability of BPCP to penetrate the tumor was assessed through intravenous injection of different formulations (free C6, BPC, and BPCP), followed by NIR-II laser (1064 nm) irradiation for 3 min. After 12 h incubation, tumors were collected, frozen sections were prepared and stained with DAPI, and the sections were fully scanned.

### The penetration depth of NIR-II Laser

Different formulations (Water, BPNSs, BP@PEG, BPS, and BPSP) were intravenously injected. After incubation for 12 h, the tumor surface was covered with 5 mm pigskin, NIR-II laser (1064 nm) irradiation for 5 min. The temperature was recorded by the camera at predetermined times.

### In vitro immunogenic cell death

The immune effector molecule related to ICD was detected in Hepa1-6 cells. Hepa1-6 cells (5 × 10^4^) were seeded on 12-well plates and incubated overnight, followed by addition of fresh medium containing PBS, Free SF, BP@PEG, or BPSP and incubation for 4 h. BP@PEG + L and BPSP + L groups were irradiated with NIR-II laser for 3 min, cultured for 4 h, and analyzed for CRT. Anti-CRT antibody was added, followed by an AF597-conjugated secondary antibody. DAPI staining and IFM were used for imaging and FCM for quantification. HMGB1 was detected by incubating cells irradiated with NIR-II laser for 12 h with anti-HMGB1 antibody for 30 min, followed by AF488-conjugated secondary antibody for 30 min. Imaging and quantification of HMGB1 release were performed using DAPI staining and an ELISA kit (Solarbio, China). ATP content was detected in the cell culture supernatant using an ATP assay kit according to the instructions.

### Antitumor efficacy in xenograft tumor model

Female C57BL/6 mice with Hepa1-6 tumors were established, and tumor inhibition was evaluated using different treatments, including BP@PEG, PD-L1 mAb, Free SF, BPSP, BP@PEG + L, BPS + L, BPP + L, and BPSP + L, with dosages of 8 mg/kg for BP and 6 mg/kg for Anti-PD-L1 and 5 mg/kg for SF. PTT treatment involved NIR-II laser irradiation (0.8 W/cm^2^, 3 min) 12 h post-administration. Mice were treated for five cycles every four days, with body weight and tumor volume monitored every other day. The formula of tumor volume was: (length × width^2^)/2. On day 21, tumors were removed, weighed, and photographed.

### Antitumor efficacy in orthotopic HCC models

The antitumor efficacy of BPSP was evaluated in vivo using an orthotopic HCC mice model with Hepa1-6-Luc tumors [34]. The mice underwent surgery to inject Hepa1-6-Luc cells into the left lobe of the liver and were randomly divided into seven treatment groups: (1) NS, (2) PD-L1 mAb, (3) BP@PEG + L, (4) Free SF, (5) Free SF + PD-L1 mAb, (6) BPSP, and (7) BPSP + L (n = 6). Treatment was injected every 4 days, with mice monitored by bioluminescence imaging every two days (Total treatment duration: 12 days). After sacrifice, the main organs were H&E-stained for preliminary safety evaluation, and livers were photographed and further analyzed.

### Statistical analysis

All data were expressed as the mean ± SD, by the Student’s t-test to analyze the statistically significant differences (GraphPad prism 8.0.3 and Microsoft Excel). *p < 0.05, **p < 0.01, ***p < 0.001.

## Results and discussion

### Preparation and characterization of BPSP

**Fig. 1:**
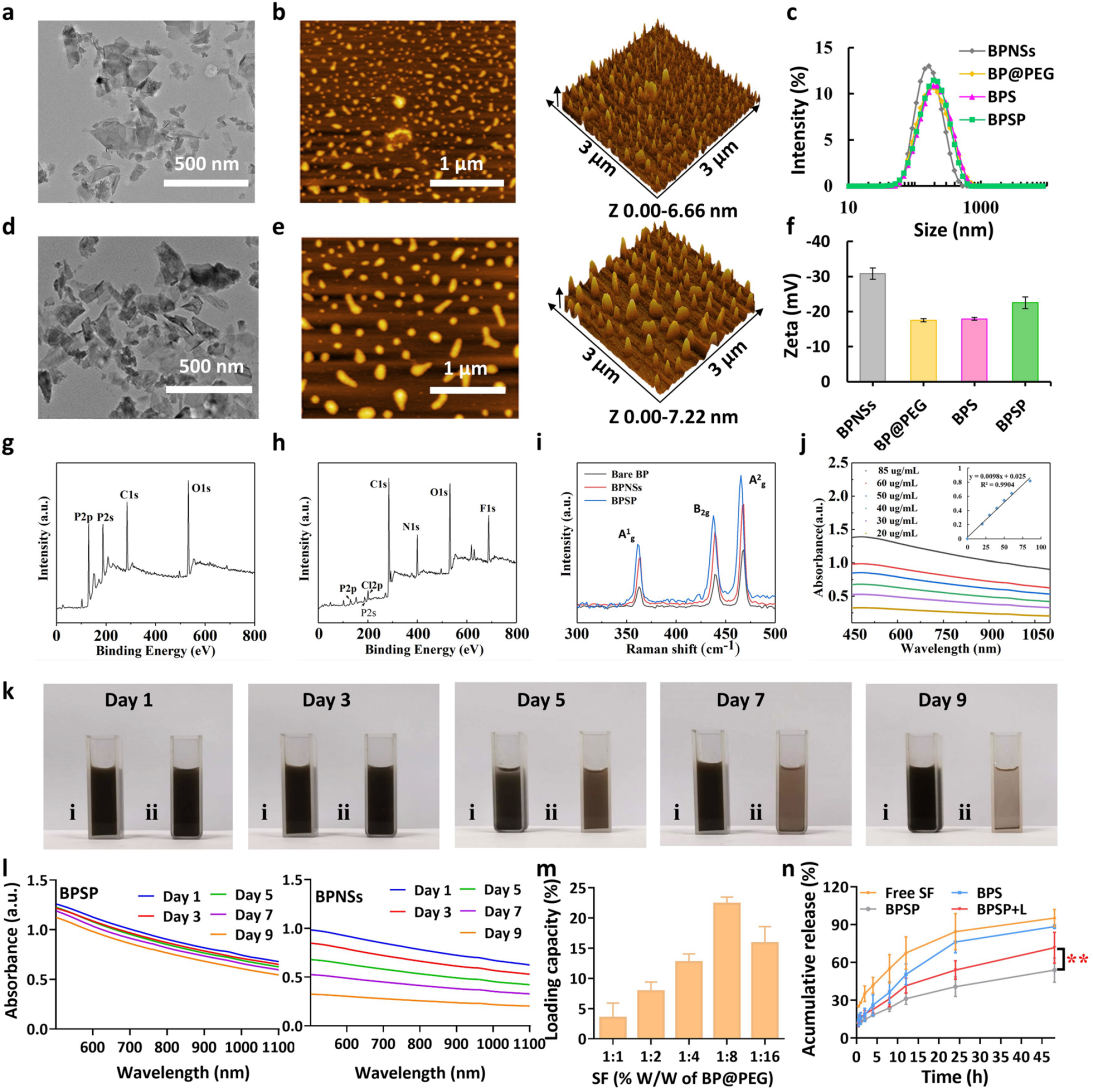
Preparation and characterization of BPSP. **a**, **b** TEM and AFM images of BPNSs, respectively. **c** Particle size of BPNSs, BP@PEG, BPS, and BPSP. **d**, **e** TEM and AFM images of BPSP. **f** Zeta potentials of BPNSs, BP@PEG, BPS, and BPSP. **g**, **h** XPS of BPNSs and BPSP. **i** Raman spectra of bulk BP and exfoliated BPNSs and BPSP. **j** Ultraviolet/visible (UV/Vis) spectra of BPNSs at different concentrations (20, 30, 40, 50, 60, and 85 μg/mL). The inset shows the normalized absorbance intensity over the characteristic cell length (A/L) at different concentrations for λ = 1064 nm. **k** Photographs of (i) BPSP and (ii) BPNSs dispersed in water after 1, 3, 5, 7, and 9 days. **l** UV/Vis spectra of (i) BPSP and (ii) BPNSs with dispersion time in the water. **m** Drug loading capacity of BP@PEG. **n** In vitro release profile of free SF, BPS, BPSP, and BPSP + L. The data are shown as the mean ± SD (n = 3). **p < 0.01

BP nanosheets (BPNSs) were synthesized through a liquid exfoliation approach, and subsequently subjected to characterization via transmission electron microscopy (TEM) and atomic force microscopy (AFM). TEM revealed that the BPNSs exhibited a sheet-like morphology and were free-standing with a lateral dimension of approximately 150 nm (Fig. 1a). The thickness of the BPNSs was about 6.66 nm based on AFM (Fig. 1b). The average diameter of BPNSs was 155.19 ± 1.93 nm (Fig. 1c and Additional file 1: Table S1). The BPSP was prepared by linking Anti-PD-L1 mAb to the surface of SF-loaded BP@PEG (BPS) via an amidation reaction. When compared with BPNSs, the size and thickness were slightly increased in BPSP. The BPSP were sheet-like with lateral sizes of about 180 nm (Fig. 1d). The thickness of BPSP was about 7.22 nm (Fig. 1e). The sizes of BP@PEG NSs, BPS, and BPSP were 162.18 ± 2.24, 165.48 ± 1.77, and 174.45 ± 3.17 nm, respectively (Additional file 1: Table S1). The zeta potential increased from − 30.83 ± 1.63 (BPNSs) to − 22.53 ± 1.64 mV (BPSP, Fig. 1f). The characteristic peak of O, C, and P emerged in the X-ray photoelectron spectroscopy (XPS) spectra of BPNSs, indicating that BPNSs are of high purity (Fig. 1g). The XPS results of BPSP, including C, O, P, Cl, and F, indicated that the BPSP was successfully prepared (Fig. 1h). Raman scattering revealed three prominent peaks at 362.57, 439, and 468.18 cm^−1^, which are attributed to three modes (A^1^_g_, B_2g_ and A^2^_g_) of bulk BP, respectively (Fig. 1i). The A^1^_g_, B_2g_, and A^2^_g_ modes of BPNSs exhibited marginal blue shifts in comparison to bulk BP, implying the efficacious exfoliation of BPNSs from the bulk form (Fig. 1i). The A^1^_g_, B_2g_, and A^2^g modes of BPSP demonstrated marginal red shifts relative to BPNSs, primarily attributable to the reduction in thickness and transverse dimension. The optical properties of BPSP were assessed via UV/Vis spectrophotometry (Fig. 1j). BPSP exhibited pronounced absorption from 450 to 1100 nm, encompassing the UV and NIR-II domains. The absorption intensities increased with the elevated concentration of BPSP. The extinction coefficient of BPSP at 1064 nm was 9.8 L g^−1^ cm^−1^. The storage stability was evaluated by exposing the aqueous suspension of BPSP to air for 9 days (Fig. 1k). BPSP retained its color, while the exposed BPNSs suspension precipitated and lightened. PEG modification effectively reduced the oxidation of BPSP, as evidenced by the absence of significant changes in its UV absorption compared to BPNSs (Fig. 1l).

To ascertain the superiority of BPNSs as a carrier, the cellular uptake of lipo@C6 and BP@PEG/C6 (BPC) was observed. BPC demonstrated significantly greater green fluorescent intensity than liposome@C6 in Hepa1-6 cells after 2 h (*p* < 0.001). This observation was validated by flow cytometry (FCM) analysis (Additional file 1: Fig. S1). These findings suggest that elongated BPNSs exhibit greater cellular uptake than spherical liposomes. The drug loading capacity reached 21.3% when the SF/BP@PEG NSs feeding ratio was 8.0 (Fig. 1m). BPSP's cumulative release significantly increased under NIR-II laser irradiation (BPSP + L) compared to BPSP alone (Fig. 1n). These results indicated that NIR-II laser irradiation expedited BPSP degradation and facilitated the release of SF.

### NIR-II-activated BPSP exhibited excellent photothermal conversion efficiency and antiangiogenic

**Fig. 2:**
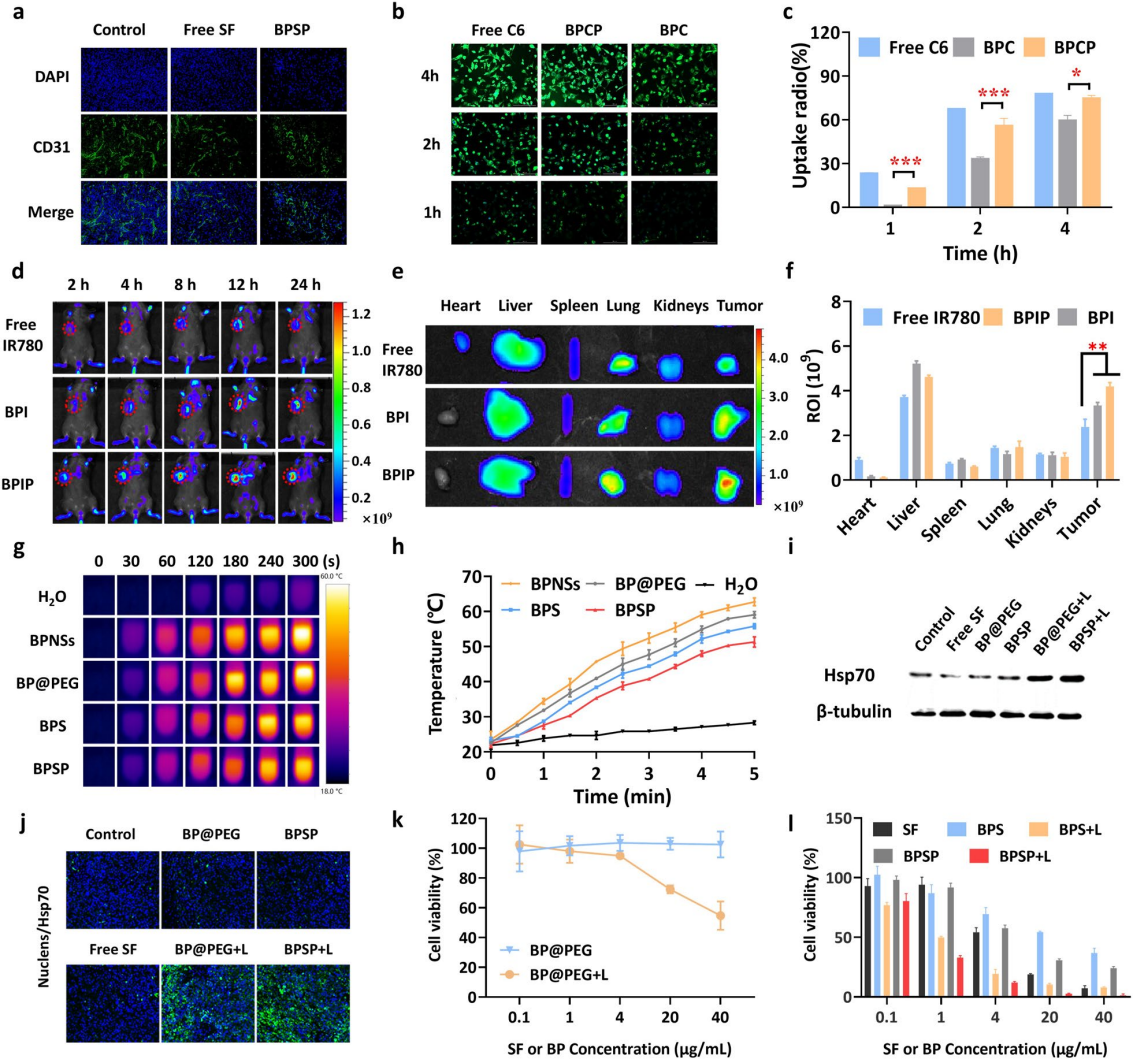
NIR-II activated BPSP impaired the growth of Hepa1-6 cells effectively with the antiangiogenic ability and specific targeting ability. **a** Blood vessels were labeled by CD31 immunofluorescence staining in subcutaneous tumors. **b**, **c** Inverted fluorescence microscope (IFM) images and FCM analysis of cellular uptake at different times. **d** Ex vivo imaging of free IR780, BPI, and BPIP in Hepa1-6 tumor-bearing mice at predetermined times. The tumor was marked with a red circle. **e** The representative images of major organs and tumors in Hepa1-6 tumor-bearing mice were observed 24 h after administration. **f** Total radiant efficiency in major organs and tumors. **g**, **h** Infrared thermal imaging and temperature profile of different concentrations under 1064 nm laser irradiation (0.8 W/cm^2^, 5 min). **i** Hsp70 expression in Hepa1-6 cells based on western blotting. **j** The expression level of Hsp70 detected by immunofluorescence staining in Hepa1-6 subcutaneous tumors treated with different groups. **k** Cell viability of Hepa1-6 cells in BP@PEG + L (0.8 W/cm^2^, 3 min) and BP@PEG groups. **l** Cell viability of Hepa1-6 cells in Free SF, BPS, BPS + L, BPSP, and BPSP + L group. Data represented mean ± SD (n = 3). *p < 0.05, **p < 0.01, ***p < 0.001, respectively

To elucidate the potential of immuno-combination therapy, we assessed the antiangiogenic and targeting abilities of BPSP. Immunofluorescence staining analysis of subcutaneous tumors revealed that the BPSP group exhibited reduced angiogenesis in comparison with the control group (Fig. 2a). We examined the C6 release of free C6 and BP@PEG/C6-αPD-L1 (BPCP) in DMEM by fluorescence spectrophotometer, the proportion of C6 released was 24% and 4.3%, respectively (Additional file 1: Fig. S2), so the C6 of BPCP was sufficient for application in the in vitro experiments. Cellular uptake experiment in different groups was assessed in Hepa1-6 cells (Fig. 2b, c). BPCP exhibited higher fluorescent intensity in Hepa1-6 cells than BPC after 2 h. Flow cytometry analysis verified this finding, with BPCP displaying a significantly higher MFI than BPC (*p* < 0.001). Tumor accumulation of BPSP was evaluated in Hepa1-6 tumor-bearing mice using IR780 as a fluorescent marker to prepare BP@PEG/IR780 (BPI) and BP@PEG/IR780-PD-L1 (BPIP). Real-time imaging showed significantly higher fluorescence intensities for the BPI and BPIP groups than the free IR780 group, indicating excellent tumor accumulation capabilities (Fig. 2d). Ex vivo imaging studies were conducted 24 h post-administration to evaluate precise distribution ability in different groups (Fig. 2e, f). The MFI of tumors in BPI and BPIP groups was significantly higher than that of the free IR780 group (*p* < 0.01 and *p* < 0.01, respectively), and BPIP showed higher tumor accumulation than BPI (*p* < 0.05). The results suggest that BPIP, guided by PD-L1 mAb, exhibits effective targeting of tumor sites, thereby facilitating the accumulation of anticancer drugs.

The photothermal capacity of different groups was assessed under NIR-II laser irradiation (1064 nm, 0.8 W/cm^2^). BPSP showed strong photothermal capacity (51.3 ℃) within 5 min (Fig. 2g, h). The temperature increase of the BPNSs solution has dose dependency (Additional file 1: Fig. S3). Photothermal stability was further evaluated under NIR-II laser (0.8 W/cm^2^) for a 5 min laser on/off cycle. The photothermal effect of the BPSP remains unchanged during the laser on and laser off-cycle, highlighting the large potential as photothermal agents of BPSP (Additional file 1: Fig. S4). Hsp70 expression during heat stress was analyzed by western blotting (Fig. 2i). After 5 min of NIR-II laser irradiation (1064 nm, 0.8 W/cm^2^), Hsp70 levels increased in the BPSP group, indicating a heat stress response in tumor cells. The in vivo tumor tissue also exhibited an enhanced Hsp70 fluorescence after BPSP treatment (Fig. 2j). The photothermal cytotoxicity of BP@PEG was first examined (Fig. 2k) after being treated with BP@PEG and NIR-II laser irradiation at 0.8 W/cm^2^ for 3 min, nearly 44% of the tumor cells survived, while the cell viability did not change in the BP@PEG group. The cytotoxicity of SF, BPS, BPSP, BPS + L, and BPSP + L in Hepa1-6 cells was then investigated. All five groups exhibited dose-dependent toxicity in Hepa1-6 cells (Fig. 2l). The IC_50_ of the BPSP + L group (0.46 ± 0.11) was higher when compared with BPSP and BPS + L groups (9.11 ± 1.93 and 0.69 ± 0.13; *p* < 0.001 and *p* < 0.01, respectively), indicating a better synergistic cytotoxicity effect of BPSP + L in vitro (Additional file 1:Table S2).

### BPSP remodels ECM by depleting collagen I and induces ICD effects

**Fig. 3:**
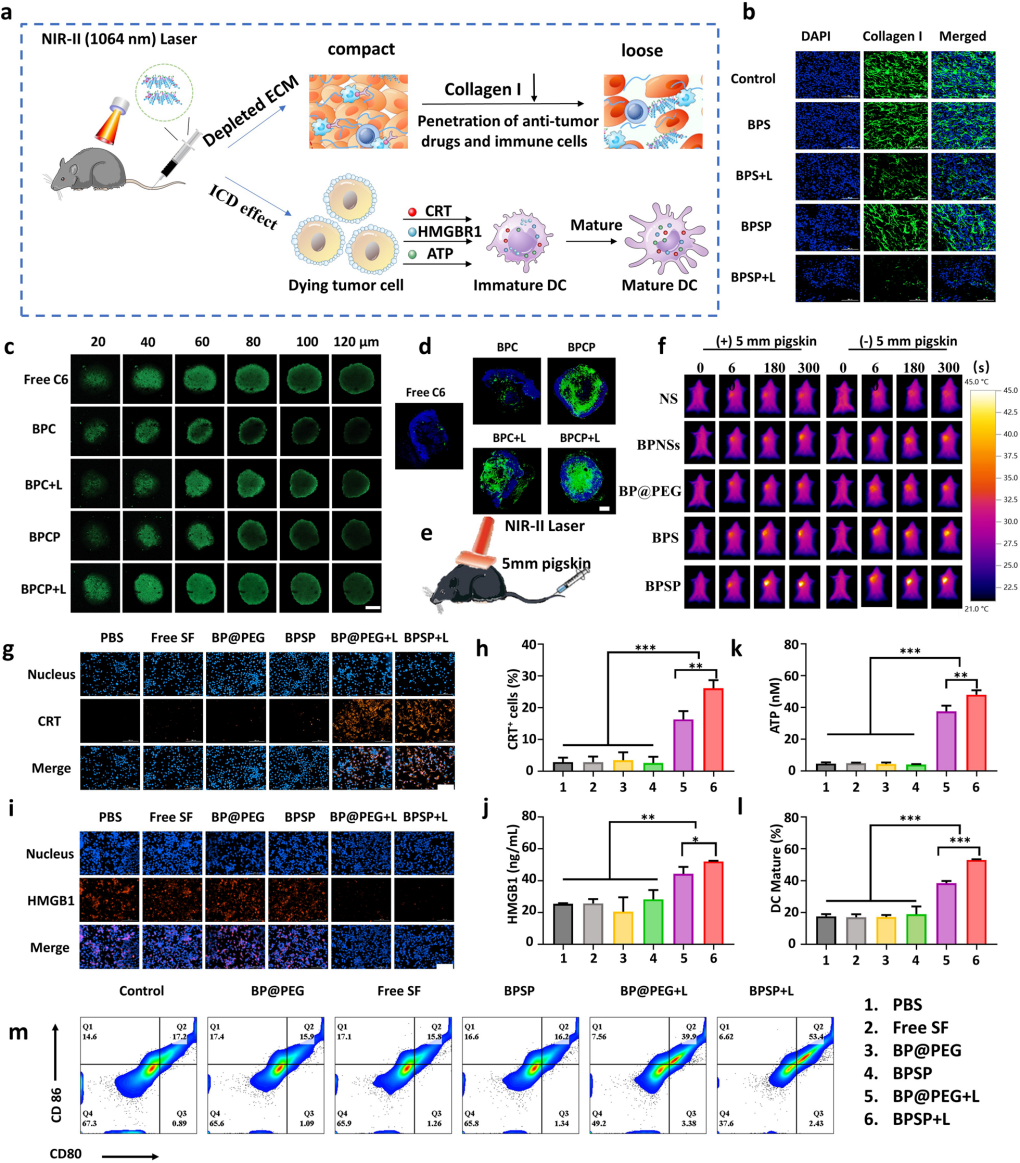
The NIR-II activated BPSP partially reduced collagen I in ECM, thus improving deep penetration, and inducing the ICD effect. **a** Schematic diagram of NIR-II activated BPSP could lead to a decrease in collagen I and promote deep penetration. **b** The expression level of collagen I in Hepa1-6 subcutaneous tumors treated with different groups. **c** Deep penetration of different groups in Hepa1-6 tumor spheroids characterized by confocal microscopy (scale bar: 200 μm). **d** Tumor tissue section after administrated of C6, BPC, and BPCP at 12 h (scale bar: 1000 μm). **e** Schematic diagram of photothermal imaging in the mice with the 5 mm pigskin. **f** Photothermal imaging of the mice between different groups with and without 5 mm pigskin by infrared thermal imaging, recorded at 0, 60, 180, and 300 s with NIR-II laser irradiation 12 h post-injection of NS, BPNSs, BP@PEG, BPS, and BPSP. **g** Fluorescence microscopy images of calreticulin (CRT) exposure in Hepa1-6 cells incubated with the different groups. **h** Fluorescence microscopy images of homo mobility group box 1 (HMGB1) exposure in Hepa1-6 cells. **i** Quantitative analysis of CRT expression capability in Hepa1-6 cells incubated with the different groups by flow cytometry. **j** Enzyme-linked immunosorbent assay (ELISA) detection of HMGB1 after treatment with the different groups in Hepa1-6 cells. **k** ATP secretion from Hepa1-6 cells. **l**, **m** DC maturation was analyzed by FCM in Hepa1-6 tumor-bearing mice. Data represented mean ± SD (n = 3). *p < 0.05, **p < 0.01, ***p < 0.001

To investigate whether NIR-II activation of BPSP caused partial depletion of ECM of HCC in vivo, the levels of collagen I in Hepa1-6 tumors were evaluated (Fig. 3b and Additional file 1: Fig. S5). The green fluorescence was significantly reduced in the BPS + L and BPSP + L groups, indicating a low level of collagen I in the ECM when exposed to NIR-II laser irradiation. The BPSP + L group showed an almost complete disappearance of collagen I, resulting in decreased ECM density and improved penetration of immune cells and antitumor drugs. Increased tumor penetration of the BPSP + L group was validated by multicellular spheroids of Hepa1-6 cells as in vitro tumor models. CLSM imaging and quantitative results showed that the BPC + L and BPCP + L groups could effectively penetrate the tumor when compared with BPC and BPCP groups in the tumor spheroids of Hepa1-6 cells (Fig. 3c and Additional file 1: Fig. S6). Furthermore, the BPCP + L group was observed in the tumor interior. In contrast, the fluorescence intensity of Free C6 + L and BPC + L was mainly distributed at the edge of the 3D tumor spheroids, but no fluorescence in the interior of the tumor interior was detected. After 12 h of administration in Hepa1-6 bearing mice, the BPCP + L group showed improved tumor penetration compared to the Free C6 group, with green fluorescence intensities mostly trapped in the tumor interior. There was almost the same in the surface temperature change of mice in different formulations when investigating the penetration depth of the NIR-II region (Fig. 3e, f). These findings suggest that NIR-II-activated BPSP can effectively consume ECM in tumors, leading to enhanced tumor penetration.

To assess the ICD effects induced by BPSP + L, we measured damage-associated molecular patterns (DAMP) expression, including calreticulin (CRT), high mobility group box 1 (HMGB1) release, and ATP secretion. IFM images showed significantly increased CRT expression (Fig. 3g) and HMGB1 release (Fig. 3h) in the BPSP + L group compared to other groups. FCM results demonstrated that the BPSP + L group had the highest CRT production at 26.14% ± 2.06% (Fig. 3i). ELISA results showed that HMGB1 release was highest in the BPSP + L group (Fig. 3j), and ATP secretion was also significantly higher in the BPSP + L group, at 48.02 ± 2.28 nm, which was 1.3-fold more than in the BP@PEG + L group (Fig. 3k). These findings consistently demonstrate that NIR-II activated BPSP has a strong photothermal capability to induce ICD effects in vitro and activate the immune response. Investigation of the ICD capabilities of different groups in vivo revealed that the BPSP + L group significantly induced CRT exposure and HMGB1 secretion, both of which were much higher than in other groups (Additional file 1: Fig. S7). Overall, NIR-II-activated BPSP demonstrated excellent ICD induction capability both in vivo and in vitro.

Dendritic cells (DCs) are essential immune cells for regulating the anti-tumor immune response. The maturation capability of the BPSP + L group to accelerate DCs was evaluated by harvesting lymph nodes after the in vivo experiment. FCM analysis showed that the BPSP + L group induced the highest DC maturation of 53.03% ± 0.40% (Fig. 3l, m). The above results indicate that BPSP + L could promote the maturation of DCs through the ICD effect.

### BPSP promoted anti-tumor immunity

**Fig. 4:**
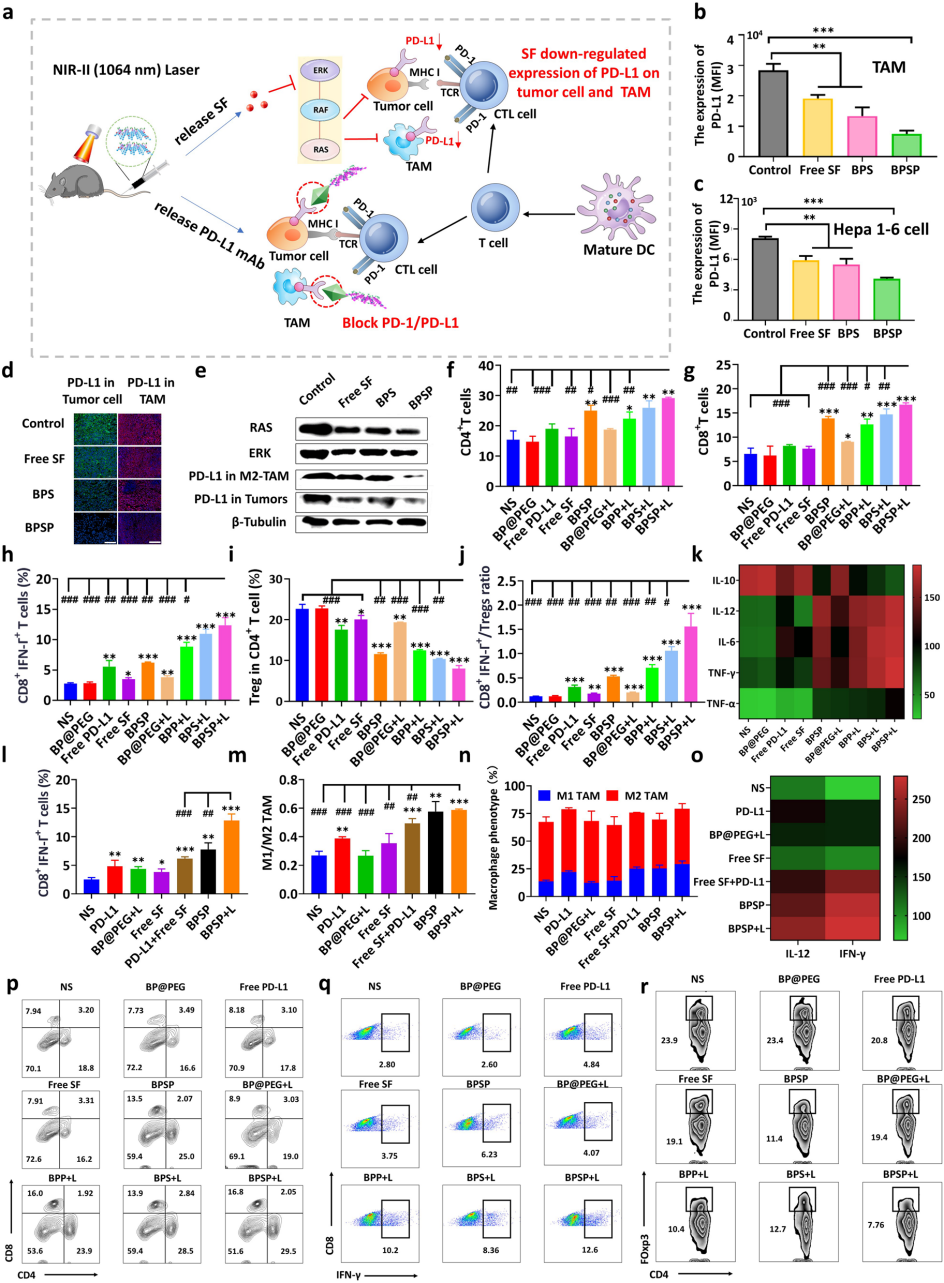
BPSP effectively caused a decrease in PD-L1 expression in Hepa1-6 cells and TAMs, improved the ratio of CTL/Tregs in tumors and promoted anti-tumor immunity. **a** Schematic diagram of reduction PD-L1 expression level in tumor cells and TAMs caused by NIR-II activated BPSP. **b** PD-L1 expression on TAMs after incubated with PBS, Free SF, BPS, and BPSP, respectively. **c** PD-L1 expression on Hepa1-6 cells after incubation with PBS, Free SF, BPS, and BPSP, respectively. **d** Immunofluorescence staining of PD-L1expression in Hepa1-6 tumors. **e** PD-L1 expression based on western blotting of Hepa1-6 cells and TAMs. **f**, **g** Quantitative analysis of CD3^+^CD4^+^CD8^+^ T cells in subcutaneous tumors based on FCM. **h**, **i** FCM analysis of infiltration ability of Tregs and CTLs in subcutaneous tumors. **j** Quantitative analysis of CD8^+^T/Tregs from subcutaneous tumors. **k** Quantitative analysis of cytokine levels (IL-6, IL-10, IL-12, IFN-γ, and TGF-α) in peripheral blood of subcutaneous mice based on ELISA. **l)** Quantitative analysis of CTLs in orthotopic tumors. **m**, **n** FCM analysis M1 TAMs and M2 TAMs in orthotopic tumors. **o** Cytokines (IL-12, IFN-γ) in peripheral blood of subcutaneous mouse measured based on ELISA Kit. **p–r** T cells, Tregs, and CTLs were quantified by flow cytometric in subcutaneous tumors. Data represented mean ± SD (n = 3). *p < 0.05, **p < 0.01, ***p < 0.001, in contrast to NS group. ^#^*p* < 0.05, ^##^*p* < 0.01, ^###^*p* < 0.001, in contrast to BPSP + L group

SF was found to inhibit PD-L1 expression in Hepa1-6 cells and TAM, which was evaluated by FCM assay in vitro (Fig. 4a–c). PD-L1 expression was significantly reduced by free SF, BPS, and BPSP, and the BPSP group had the lowest expression level. Immunofluorescence of tumor tissues and western blotting results confirmed the same conclusion. BPSP could suppress PD-L1 expression in tumor cells and TAMs by inhibiting the RAS/RAF/ERK signaling pathway.

Inhibition of PD-L1 expression led to an improvement in the CTL/Tregs ratio in the tumor site and improved the efficacy of immune-combination therapy. The abundance of immune cells and cytokines in the tumor site was estimated to confirm this (Fig. 4f–r). The frequency of CD3^+^CD4^+^ T cells and CD3^+^CD8^+^ T cells in the BPSP + L group was significantly higher than the NS group and other groups (*p* < 0.01 and *p* < 0.001, respectively), indicating effective intra-tumor infiltration of T cells. CD8^+^IFN-γ^+^ CTLs proportion in the BPSP + L group was much higher than other groups (*p* < 0.001), while the Tregs proportion was much lower, implying that NIR-II activated BPSP could enhance the CTL/Treg ratio. The ratio of CD8^+^/Tregs in the BPSP + L group was also higher than in other groups, which is a key index for evaluating the immune effects of anti-tumor therapy. Furthermore, The BPSP + L group effectively suppressed IL-10 and TGF-β levels (*p* < 0.001 and *p* < 0.001, respectively) while exhibiting the highest expression of INF-γ, IL-6, and 12, and TNF-α compared to the NS group (Fig. 4k and Additional file 1: Fig. S8), indicating the potential of BPSP + L to promote anti-tumor immune response by regulating immune cytokines. Immune cell abundance (CD8^+^IFN-γ^+^ CTLs, TAMs) and immune cytokines (INF-γ, IL-12) were measured in the orthotopic HCC model. The BPSP + L had significantly higher levels of CTLs than the free SF + PD-L1 group (*p* < 0.001) and other groups, indicating increased infiltration of CTLs to TIME (Fig. 4l). The M1/M2 ratio was also enhanced, suggesting efficient repolarization of M2 TAMs to M1 TAMs (Fig. 4m, n). The BPSP + L had the highest levels of INF-γ and IL-12, and the lowest level of TGF-β (Fig. 4o and Additional file 1: Fig. S9), further demonstrating the strengthening of the CTL/Tregs ratio and relief of suppressive TIME by BPSP + L.

### NIR-II activated BPSP enhanced antitumor efficacy in subcutaneous tumor model

**Fig. 5:**
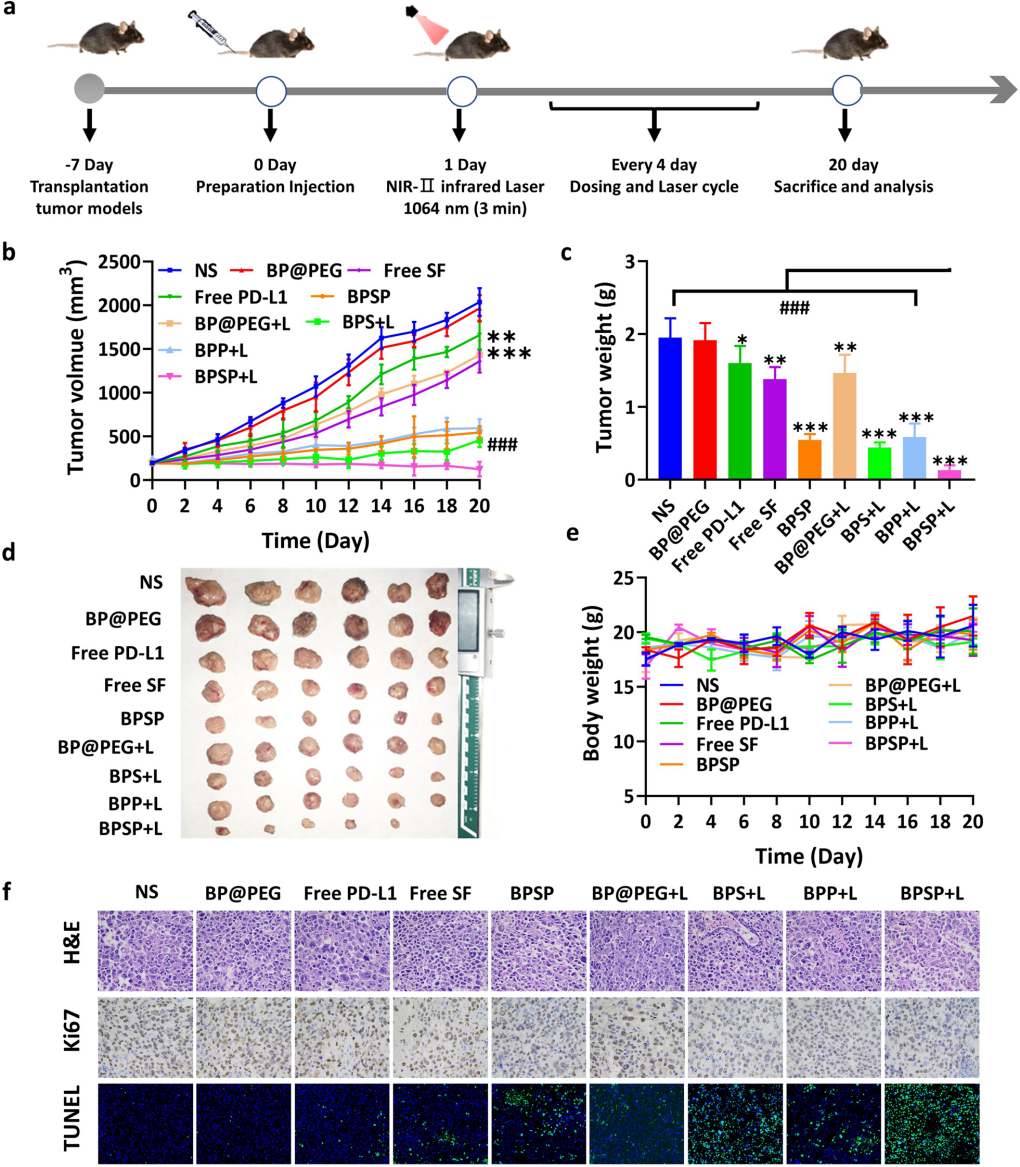
Evaluation of the antitumor therapeutic effects of NIR-II activated BPSP in a subcutaneous tumor model. **a** Scheme of NIR-II activated BPSP in a subcutaneous tumor model. **b** Tumor growth curves of tumor-bearing mice in different groups during treatment. **c**, **d** Tumor weight and photographs of different groups after treatment. **e** Body weight changes during treatment. **f** Immunohistochemical sections of tumor (H&E ×400, Ki67 ×400, TUNEL ×50). Data represented mean ± SD (n = 6). *p < 0.05, **p < 0.01, ***p < 0.001, compared with the NS group. ^###^*p* < 0.001, compared with the BPSP + L group

The antitumor efficacy of NIR-II activated BPSP was investigated in C57BL/6 mice with Hepa1-6 subcutaneous tumor, and the volume was monitored every two days (Fig. 5a–d, Fig. S10). From the results we can observe that there was no significant difference between NS and BP@PEG groups, it suggested that BP@PEG is safe and non-toxic. The anti-tumor ability of the free PD-L1 group is weaker compared with the free SF and BP@PEG + L group, with the anti-tumor ability in the order free SF≈BP@PEG + L > free PD-L1 group, showing single immunotherapy has little effect. Of note, after 3 weeks treatment, compared with a single drug, BPS + L, BPP + L, and BPSP groups showed expected excellent effects, which fully demonstrates the advantages of combination. Treatment with BPS + L, BPP + L, BPSP, and BPSP + L groups inhibited all tumor growth in vivo even could not completely ablate tumor progression. As expected, the BPSP + L treatment group had the smallest tumor volume and the highest tumor growth inhibition rate (93.32%, Additional file 1: Table S3). Taken together, our results indicated that the photothermal-boosted-NanoBike plays a photothermal/chemical/immune synergistic therapeutic effect. Meanwhile, BPSP + L produced low systemic toxicity as indicated by no significant change in body weight during the experiment (Fig. 5e).

Hemocompatibility of BPSP was confirmed by investigating hemolysis capacity, which showed a low hemolysis rate (Additional file 1: Fig. S11). H&E, Ki67, and TUNEL staining revealed that BPSP + L induced the highest degree of cell necrosis, the least proliferation of tumor cells, and the most apoptosis, indicating robust antitumor activity (Fig. 5e). No damage to main organs was observed in any group, demonstrating the safety of the BPSP + L group (Additional file 1: Fig. S12). In short, these results indicated that BPSP + L manifests excellent antitumor capability and higher safety.

### NIR-II activated BPSP enhanced antitumor efficacy in orthotopic HCC model

**Fig. 6:**
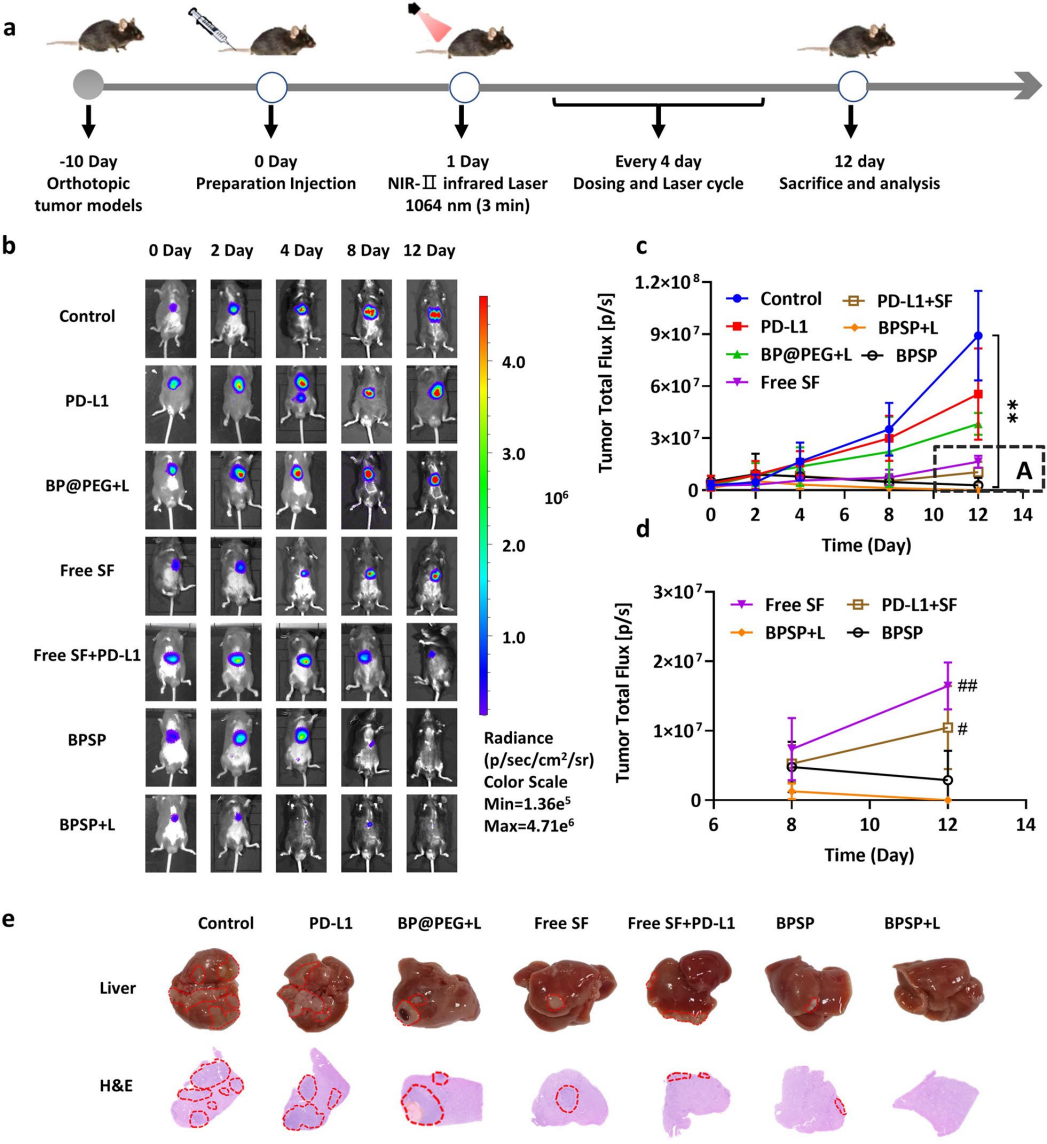
NIR-II activated BPSP robust antitumor efficacy in orthotopic HCC models. **a** Schematic in orthotopic HCC models. **b** Bioluminescence images of tumor-bearing mice in different groups during treatment. **c** Bioluminescence intensity curves of tumor-bearing mice. **d** Bioluminescence intensity curves of tumor-bearing mice in A area. **e** Representative photographs and H&E results of the liver with each group. Data represented mean ± SD (n = 3). **p < 0.01, in contrast to the NS group. ^##^*p* < 0.01, ^#^*p* < 0.0, in contrast to BPSP + L group

The antitumor effect of NIR-II activated BPSP was measured in orthotopic tumor-bearing mice by monitoring tumor growth through bioluminescence imaging (Fig. 6a). In this model, the free SF + PD-L1 group is added in order to simulate the “T + A” scheme (the combination of atezolizumab and bevacizumab), it has shown encouraging clinical benefits by targeting both angiogenesis and PD-L1 signaling in hepatocellular carcinoma (HCC) treatment. The bioluminescence intensity of the free PD-L1 and BP@PEG + L groups did not significantly differ compare with the NS group- is different from that of the subcutaneous tumor model, it may be caused by TIME and the larger tumor depth in vivo. A significant decrease was observed in the free SF, free SF + PD-L1, BPSP, and BPSP + L groups (Fig. 6b). Notably, the BPSP + L group exhibited almost no bioluminescence intensity on day 12 (Fig. 6c), and its intensity was found to be the weakest among all treatment groups (Fig. 6d), the experimental results were consistent with the subcutaneous tumor. Only on the 2nd day, the bioluminescence intensity of free SF + PD-L1 and BPSP + L was significantly lower than NS group, and the BPSP + L group exhibited a lower intensity than the free SF + PD-L1 group (p < 0.05). These findings support the strong antitumor effect of the BPSP + L group in the orthotopic HCC model and made an improvement based on the “T + A” scheme. The results of H&E staining were consistent with photographs of the liver, indicating that the BPSP + L group had almost no tumor volume in the liver (Fig. 6e). No pathological changes were observed in major organs, confirming the desired safety of the BPSP + L group in the orthotopic HCC mice model (Additional file 1: Fig. S13). In brief, the BPSP + L group exhibited robust antitumor activity and desired safety in the orthotopic model.

## Conclusions

In light of the crucial role of immune escape by tumor cells, tumor angiogenesis, and ECM, a novel self-delivery photothermal-boosted-nanoformulation was developed involving NIR-II activated BP tandem-augmented Anti-PD-L1 mAb plus SF to remodel the TIME and convert "cold" tumors into "hot" ones for achieving optimal ICIs therapy efficacy. This PTT-induced treatment led to a partial reduction in collagen I within the ECM, promoting deep infiltration of immune cells and antitumor drugs, boosting SF release to exert antivascular ability and suppress PD-L1 expression on tumor cells and TAMs through the RAS/RAF/ERK pathway, inducing the ICD effect. Notably, NIR-II-activated BPSP exhibited the most significant inhibition of tumor growth in both subcutaneous and orthotopic HCC models, indicating enhanced responsiveness to ICIs-combination therapy. These findings suggest a promising approach for immuno-combination therapy that can be applied to treat various types of cancer in clinical settings.

### Supplementary Information

**Additional file 1:** **Figure S1**. a-b) IFM images and FCM analysis of cellular uptake of C6 loaded Liposome and BP@PEG/SF in Hepa1-6 cells in 2 h. Data were shown as mean ± SD (n=3). **p < 0.01. **Figure S2.** *In vitro* release profile of free C6, BPCP. **Figure S3.** Photothermal properties of BPNSs at varied concentrations. **Figure S4.** The heating curve of different concentrations dispersed in water for four cycles at a power intensity of 0.8 W/cm^−2^ under irradiation by 1064 nm laser. **Figure S5.** Quantification of the mean arbitrary units (AU) with Image J software. **Figure S6.** Quantification of the mean fluorescence intensity (MFI) with ZEN software and the intensity was normalized to the highest MFI. Data were shown as mean ± SD (n=3). ***p < 0.001. **Figure S7.** a-b) Immunofluorescence staining of CRT exposure and HMGB1 release in Hepa1-6 tumors. **Figure S8.** Cytokines in blood serum. a IL-10, b TGF-β, c IL-12, d IL-6, e IFN-γ, f TNF-α. *p < 0.05, **p < 0.01, ***p < 0.001, compared with NS group. **Figure S9**. Cytokines in blood serum. a IL-12, b IFN-γ, c TGF-β. *p < 0.05, **p < 0.01, ***p < 0.001, compared with NS group. **Figure S10. **Tumor growth curves of different groups. a NS, b BP@PEG, c Free PD-L1, d Free SF, e BPSP, f BP@PEG+L, g BPS+L, h BPP+L, i BPSP+L. **Figure S11.** Hemolysis assays of BPSP. a Photograph of hemolysis samples for BPSP. b HR% of BPSP at different concentrations.Sample“-”: NS group; Test-tube 1-5: 5, 15, 25, 50, 75 ug/mL of BPSP; Sample“+”: Positive control (Water). **Figure S12.** Immunohistochemical analysis of H&E-stained sections after treatment in subcutaneous model (scale bar=400×). **Figure S13**. Immunohistochemical analysis of H&E-stained sections after treatment in orthotopic model (scale bar=400×). **Table S1**. Size, PDI and zeta potential of BPSP (data represent mean ± SD, n = 3). **Table S2.** IC50 in different treatment group.**Table S3. **Tumor inhibition rates of different treatment groups.

## Data Availability

All data are available in the main text and are available from the corresponding authors upon reasonable request.
