# Supplementary material for: Self-delivery photothermal-boosted-nanobike multi-overcoming immune escape by photothermal/chemical/immune synergistic therapy against HCC
Source: J Nanobiotechnology. 2024 Mar 29;22:137. doi: 10.1186/s12951-024-02399-3 (PMC10981284; doi:10.1186/s12951-024-02399-3)
Supplement: Supplementary file 1 — Additional file 1: Figure S1. a-b) IFM images and FCM analysis of cellular uptake of C6 loaded Liposome and BP@PEG/SF in Hepa1-6 cells in 2 h. Data were shown as mean ± SD (n=3). **p < 0.01. Figure S2. In vitro release profile of free C6, BPCP. Figure S3. Photothermal properties of BPNSs at varied concentrations. Figure S4. The heating curve of different concentrations dispersed in water for four cycles at a power intensity of 0.8 W/cm−2 under irradiation by 1064 nm laser. Figure S5. Quantification of the mean arbitrary units (AU) with Image J software. Figure S6. Quantification of the mean fluorescence intensity (MFI) with ZEN software and the intensity was normalized to the highest MFI. Data were shown as mean ± SD (n=3). ***p < 0.001. Figure S7. a-b) Immunofluorescence staining of CRT exposure and HMGB1 release in Hepa1-6 tumors. Figure S8. Cytokines in blood serum. a IL-10, b TGF-β, c IL-12, d IL-6, e IFN-γ, f TNF-α. *p < 0.05, **p < 0.01, ***p < 0.001, compared with NS group. Figure S9. Cytokines in blood serum. a IL-12, b IFN-γ, c TGF-β. *p < 0.05, **p < 0.01, ***p < 0.001, compared with NS group. Figure S10. Tumor growth curves of different groups. a NS, b BP@PEG, c Free PD-L1, d Free SF, e BPSP, f BP@PEG+L, g BPS+L, h BPP+L, i BPSP+L. Figure S11. Hemolysis assays of BPSP. a Photograph of hemolysis samples for BPSP. b HR% of BPSP at different concentrations.Sample“-”: NS group; Test-tube 1-5: 5, 15, 25, 50, 75 ug/mL of BPSP; Sample“+”: Positive control (Water). Figure S12. Immunohistochemical analysis of H&E-stained sections after treatment in subcutaneous model (scale bar=400×). Figure S13. Immunohistochemical analysis of H&E-stained sections after treatment in orthotopic model (scale bar=400×). Table S1. Size, PDI and zeta potential of BPSP (data represent mean ± SD, n = 3). Table S2. IC50 in different treatment group.Table S3. Tumor inhibition rates of different treatment groups. [file 12951_2024_2399_MOESM1_ESM.docx]

**Self-delivery Photothermal-Boosted-NanoBike Multi-Overcoming Immune Escape by Photothermal/chemical/immune Synergistic Therapy against HCC**

Huizhen Yang#, Weiwei Mu#, Shijun Yuan, Han Yang, Lili Chang, Xiao sang, Tong Gao, Shuang Liang, Xiaoqing Liu, Shunli Fu, Zipeng Zhang, Yongjun Liu*, Na Zhang*

#These authors contributed equally to this work.

NMPA Key Laboratory for Technology Research and Evaluation of Drug Products, Department of Pharmaceutics, Key Laboratory of Chemical Biology (Ministry of Education), School of Pharmaceutical Sciences, Cheeloo College of Medicine, Shandong University, 44 Wenhua Xi Road, Jinan, Shandong Province 250012, China

**Additional file**

**Materials and Methods**

**1. Preparation of Liposomes@C6.** Liposomes containing Coumarin-6 (C6) were prepared via film dispersion method using EPC (40 mg), Chol (5 mg), and C6 (1 mg) dissolved in 2 ml ethanol, evaporated at 40℃ to form a dry lipid film, and then hydrated with 0.001 M PBS (pH 7.4), followed by extrusion through 0.22 μm and 0.20 μm membrane filters using a LiposoFast-Basic extruder (Avestin, Ottawa, ON, Canada).

**2. Comparison of carrier superiority.** C6 was used as a label for preparing BP@PEG/C6 (BPC) and Liposome@C6. Hepa1-6 cells (2 × 10^5^ cells/well) were seeded in 12-well plates and exposed to BP@PEG/C6 and Liposome@C6 solutions containing 200 ng/mL C6. After a 2-hour incubation period, cells were trypsinized and analyzed using FCM.

**3. Immunohistochemical analysis and Hemolysis experiment.** Tumors and main organs were resected and fixed with 4% paraformaldehyde for histological analysis using H&E, Ki67, and TUNEL staining sections, while H&E staining sections were used to observe organs for the safety experiment. BPSP at varying concentrations was incubated with 2% RBC at 37°C for 3 hours, and the resulting hemoglobin absorbance was determined by UV/Vis at 576 nm.

**
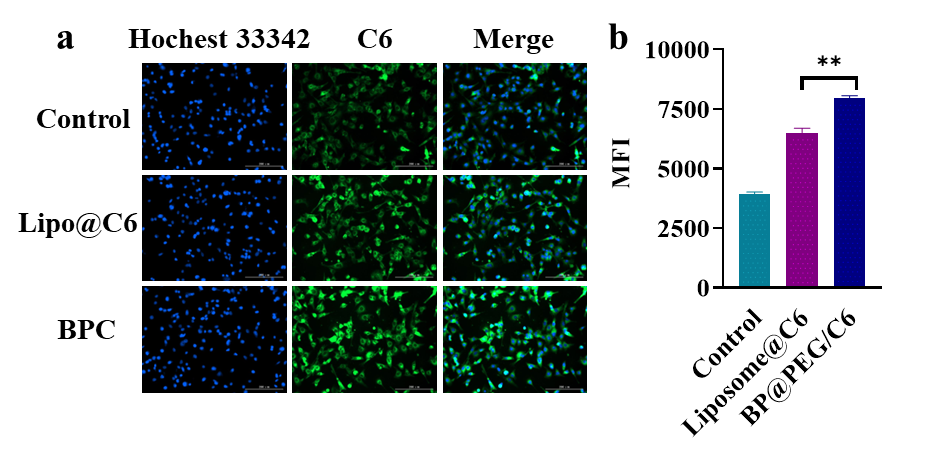
**

**Figure S1.** a-b) IFM images and FCM analysis of cellular uptake of C6 loaded Liposome and BP@PEG/SF in Hepa1-6 cells in 2 h. Data were shown as mean ± SD (n=3). ***p* < 0.01.


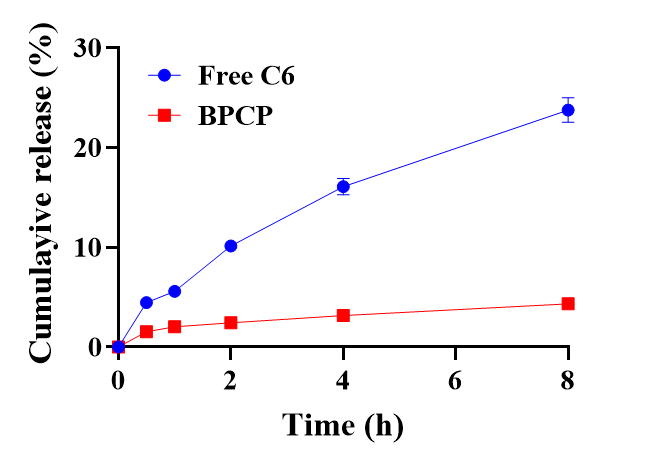


**Figure S2**. *In vitro* release profile of free C6, BPCP.

**Figure S3.** Photothermal properties of BPNSs at varied concentrations.

**Figure S4.** The heating curve of different concentrations dispersed in water for four cycles at a power intensity of 0.8 W/cm^−2^ under irradiation by 1064 nm laser.


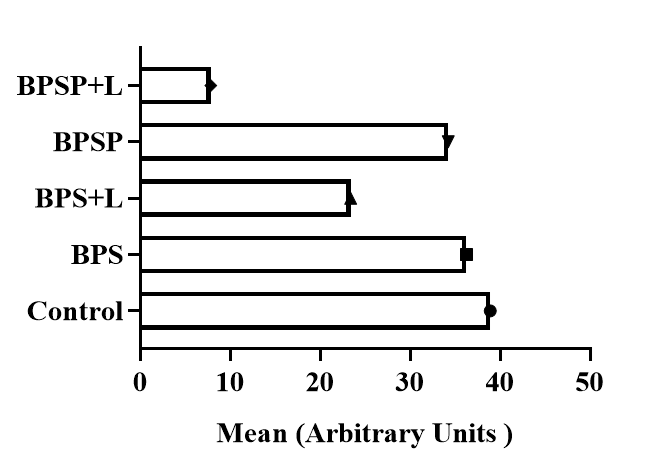


**Figure S5.** Quantification of the mean arbitrary units (AU) with Image J software.

**Figure S6.** Quantification of the mean fluorescence intensity (MFI) with ZEN software and the intensity was normalized to the highest MFI. Data were shown as mean ± SD (n=3). ****p* < 0.001.

**
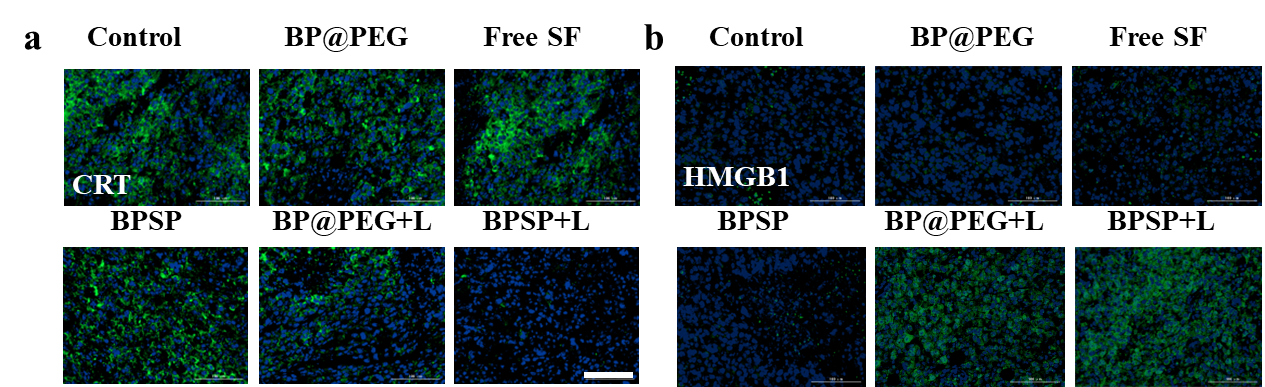
**

**Figure S7.** a-b) Immunofluorescence staining of CRT exposure and HMGB1 release in Hepa1-6 tumors.

**
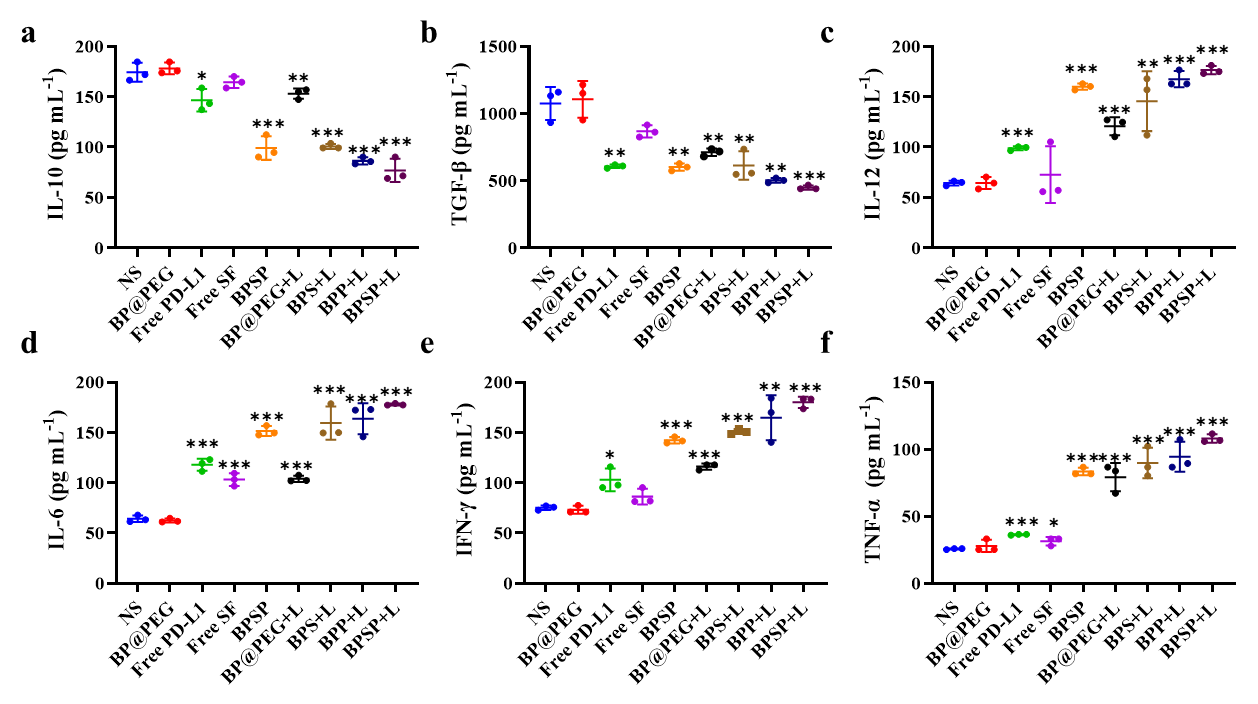
**

**Figure S8.** Cytokines in blood serum. **a** IL-10, **b** TGF-β, **c** IL-12, **d** IL-6, **e** IFN-γ, **f** TNF-α. **p* < 0.05, ***p* < 0.01, ****p* < 0.001, compared with NS group.

**
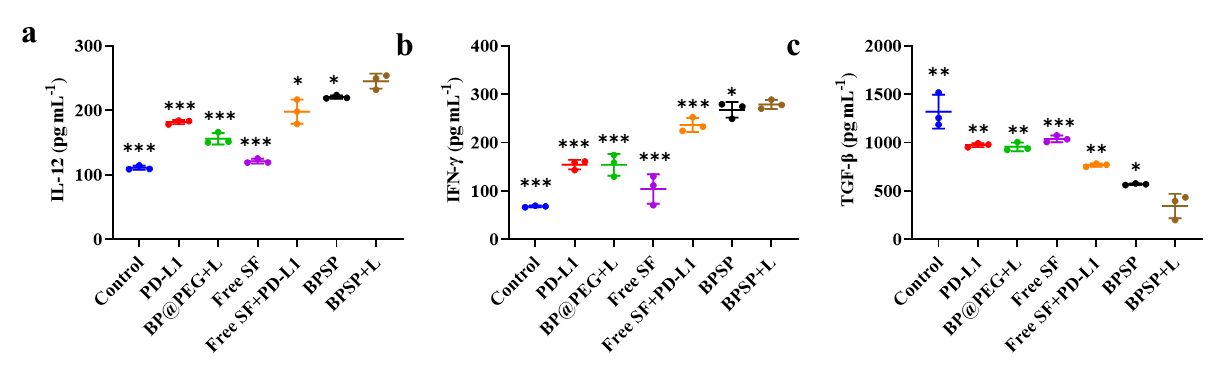
**

**Figure S9.** Cytokines in blood serum. **a** IL-12, **b** IFN-γ, **c** TGF-β. **p* < 0.05, ***p* < 0.01, ****p* < 0.001, compared with NS group.

**
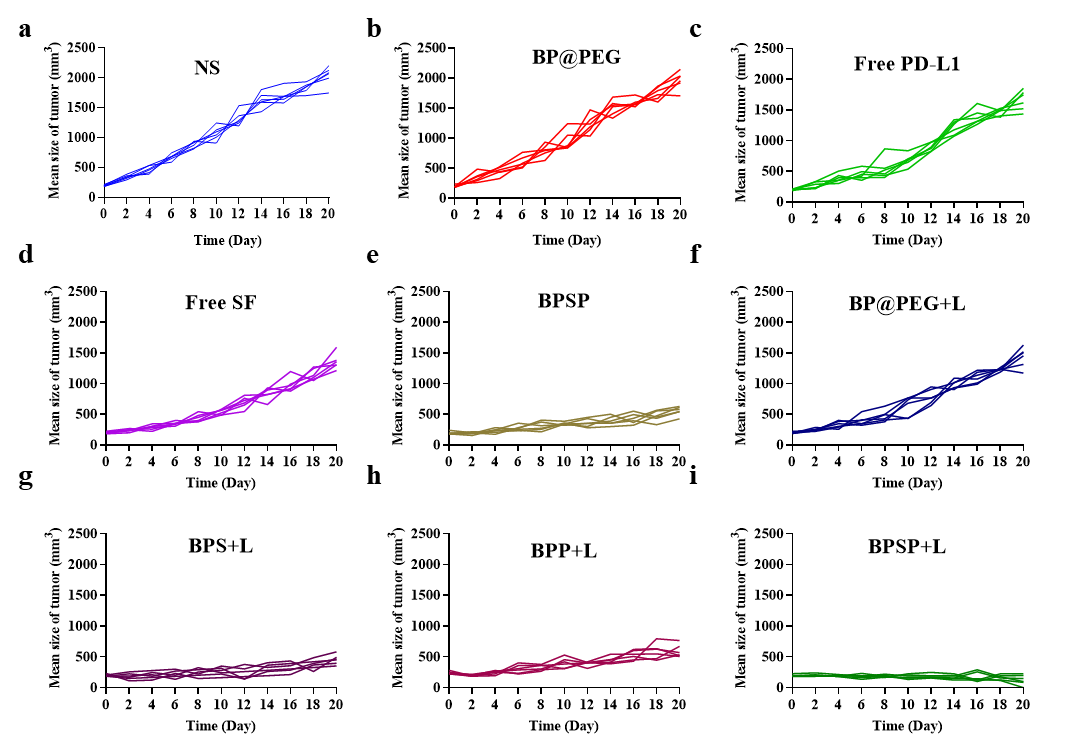
**

**Figure S10.** Tumor growth curves of different groups. **a** NS, **b** BP@PEG, **c** Free PD-L1, **d** Free SF, **e** BPSP, **f** BP@PEG+L, **g** BPS+L, **h** BPP+L, **i** BPSP+L.


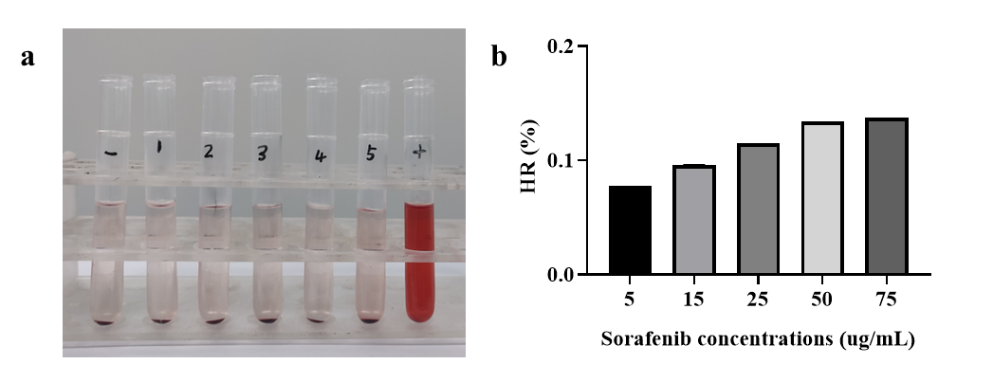


**Figure S11.** Hemolysis assays of BPSP. **a** Photograph of hemolysis samples for BPSP. **b** HR% of BPSP at different concentrations.Sample“-”: NS group; Test-tube 1-5: 5, 15, 25, 50, 75 ug/m

L of BPSP; Sample“+”: Positive control (Water).


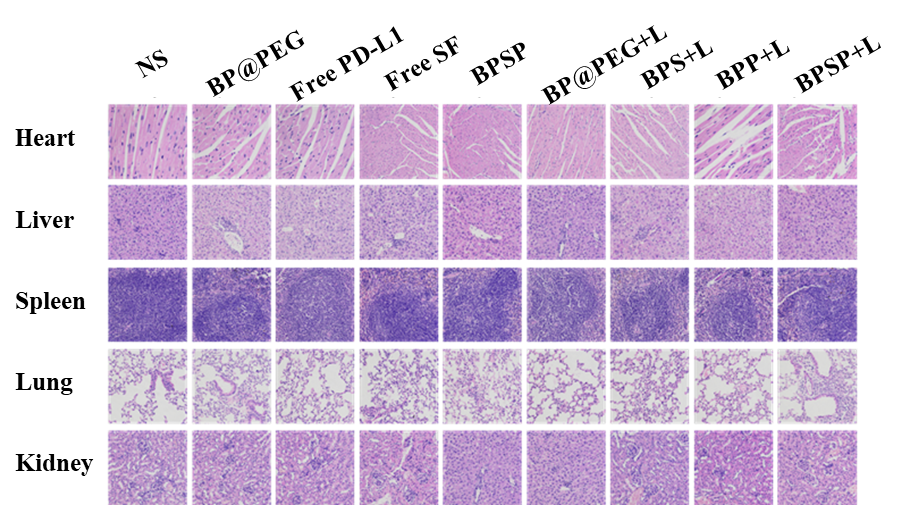


**Figure S12.** Immunohistochemical analysis of H&E-stained sections after treatment in subcutaneous model (scale bar=400×).


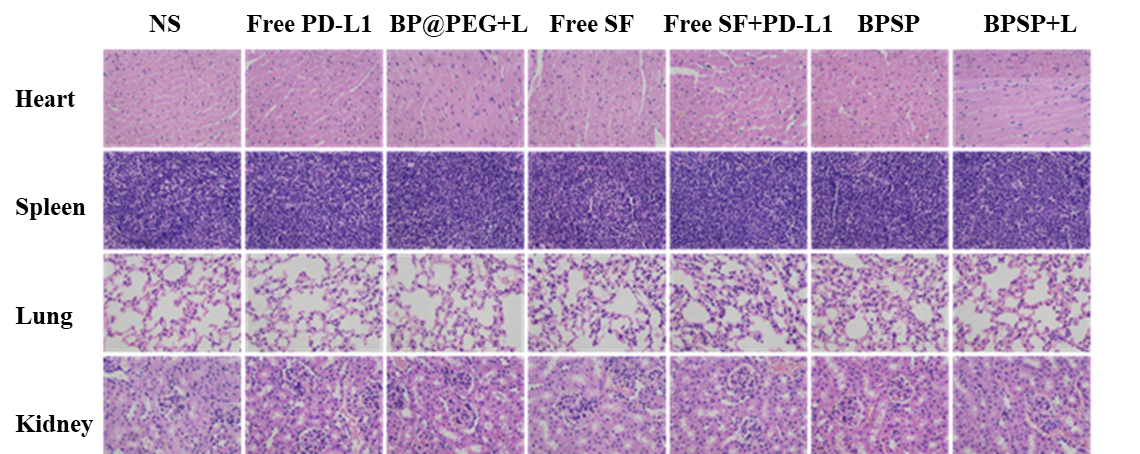


**Figure S13.** Immunohistochemical analysis of H&E-stained sections after treatment in orthotopic model (scale bar=400×).

**Table S1.** Size, PDI and zeta potential of BPSP (data represent mean ± SD, n = 3).

| Groups | Average size  ±SD (nm) | Average PDI  ±SD | Average zeta  ±SD (mv) | Sorafenib (DL%) | PD-L1 mAb  (DL%) |
| --- | --- | --- | --- | --- | --- |
| BPNSs | 155.19±1.93 | 0.30±0.10 | -30.83±1.63 | / | / |
| BP@PEG | 162.18±2.24 | 0.28±0.01 | -17.53±0.45 | / | / |
| BPS | 165.48±1.77 | 0.26±0.02 | -17.93±0.42 | 9.27±2.92 | / |
| BPSP | 174.45±3.17 | 0.21±0.12 | -22.53±1.64 | 8.29±2.66 | 10.59±0.53 |

**Table S2.** IC_50_ in different treatment group.

| Group | SF | BPS | BPS+L | BPSP | BPSP+L |
| --- | --- | --- | --- | --- | --- |
| IC_50_ (μg/mL) | 5.53±2.82 | 21.87±3.20 | 0.69±0.13 | 9.11±1.93 | 0.46±0.11 |

**Table S3.** Tumor inhibition rates of different treatment groups.

| Groups | Tumor inhibition rate (%) |
| --- | --- |
| Free PD-L1 | 25.62 |
| Free SF | 38.89 |
| BPSP | 75.52 |
| BP@PEG+L | 35.68 |
| BPP+L | 73.24 |
| BPS+L | 79.53 |
| BPSP+L | 93.32 |
